# Arabinogalactan proteins are involved in root hair development in barley

**DOI:** 10.1093/jxb/eru475

**Published:** 2014-12-01

**Authors:** Marek Marzec, Iwona Szarejko, Michael Melzer

**Affiliations:** ^1^Department of Genetics, Faculty of Biology and Environmental Protection, University of Silesia, Katowice 40-032, Poland; ^2^Department of Physiology and Cell Biology, Leibniz Institute of Plant Genetics and Crop Plant Research (IPK), Gatersleben D-06466, Germany

**Keywords:** Arabinogalactan proteins (AGPs), barley (*Hordeum vulgare*), cell differentiation, monoclonal antibodies, root hairs, Yariv.

## Abstract

The arabinogalactan proteins (AGPs) are involved in a range of plant processes, including cell differentiation and expansion. Here, barley root hair mutants and their wild-type parent cultivars were used, as a model system, to reveal the role of AGPs in root hair development. The treatment of roots with different concentrations of βGlcY (a reagent which binds to all classes of AGPs) inhibited or totally suppressed the development of root hairs in all of the cultivars. Three groups of AGP (recognized by the monoclonal antibodies LM2, LM14, and MAC207) were diversely localized in trichoblasts and atrichoblasts of root hair-producing plants. The relevant epitopes were present in wild-type trichoblast cell walls and cytoplasm, whereas in wild-type atrichoblasts and in all epidermal cells of a root hairless mutant, they were only present in the cytoplasm. In all of cultivars the higher expression of LM2, LM14, and MAC207 was observed in trichoblasts at an early stage of development. Additionally, the LM2 epitope was detected on the surface of primordia and root hair tubes in plants able to generate root hairs. The major conclusion was that the AGPs recognized by LM2, LM14, and MAC207 are involved in the differentiation of barley root epidermal cells, thereby implying a requirement for these AGPs for root hair development in barley.

## Highlight

Arabinogalactan proteins (AGPs) recognized by the LM2, LM14, and MAC207 antibodies are diversely deposited in rhizodermis of barley root hair-producing genotypes, but evenly deposited in rhizodermal cells of the root hairless mutant.

## Introduction

The arabinogalactan proteins (AGPs) are a large heterogeneous family of hydroxyproline-rich glycoproteins found both within and on the surface of plant cells (Fincher *et al.*, 1983; Nguema-Ona *et al.*, 2012), and in representatives of the entire plant kingdom, including mosses (Lee *et al.*, 2005). They are involved in a large number of biological processes, including cell division (Langan and Nothnagel, 1997), programmed cell death (Gao and Showalter, 1999; Guan and Nothnagel, 2004), cell differentiation (Majewska-Sawka and Nothngel, 2000; dos Santos *et al.*, 2006), cell expansion (Darley *et al.*, 2001; Lu *et al.*, 2001) and host/microbe interactions (van Buuren *et al.*, 1999; Johnson *et al.*, 2003; Nguema-Ona *et al.*, 2013). Some AGPs are directed to the cytosol, and some others to the extracellular matrix (Youl *et al.*, 1998); they typically attach to the plasma membrane by means of a glycosylphosphatidylinositol (GPI) anchor. Other AGPs are secreted into either the intercellular space (Samaj *et al.*, 2000) or to the plant’s exterior in the form of mucilage (Moody *et al.*, 1988). Although their molecular size can vary from 60 to 300kDa, they all consist of a short peptide core surrounded by carbohydrate moieties which comprise at least 90% of the molecule’s mass (Serpe and Nothnagel, 1999). The glycan part consists of sugars (arabinose, galactose, rhamnose, fucose, glucuronic acid, and xylose) (Nothnagel, 1997; Showalter, 2001) generating a carbohydrate moiety which varies greatly both between species, and even between organs of a given species (Tsumuraya *et al.*, 1988; Pennell *et al.*, 1991; Seifert and Roberts, 2007). The form of post-translational modification of the AGPs may influence their function more strongly than does their peptide sequence (Nguema-Ona *et al.*, 2012).

An important tool for AGP investigation is a Yariv reagent. The reactive form, containing β-D-glucosyl residues (βGlcY), is capable of binding and/or precipitating AGPs (Yariv *et al.*, 1962). Plants or organs treated with a Yariv reagent are deprived of functional AGPs naturally present on their surface, which allows their functional investigation *in vivo*. However, βGlcY binds to all AGPs, what prevents its application for analyses of individual classes of AGPs (Yariv *et al.*, 1962; Paulsen *et al.*, 2014). For more detailed investigations of AGPs, monoclonal antibodies (mAbs) recognizing specific epitopes associated with the carbohydrate moieties have been used (Nguema-Ona *et al.*, 2012).

The role of AGPs during root development and morphogenesis was shown using the active form of Yariv reagent. Exposure of the *Arabidopsis thaliana* root to βGlcY suppresses the elongation of epidermal cells and hence reduces root growth (Willats and Knox, 1996). AGPs are known to influence the organization of cortical microtubules, which control the elongation of epidermal cells (Nguema-Ona *et al.*, 2007). Periplasmic AGPs can also act as calcium capacitors, which is significant because calcium ion gradients are important for cell expansion (Lamport and Varnai, 2012). In the barley (*Hordeum vulgare*) root hairless mutant *rhl.1a*, an *HvAGP* gene was upregulated by four orders of magnitude compared to the wild-type level, but there was no such upregulation in a second mutant (*rhp1.a*) which developed root hairs unable to progress beyond the primordium stage (Kwasniewski *et al.*, 2010). There is no more evidence about the role of AGPs in root hair development, although the pollen tube as another cell expressing tip growth was extensively studied in this context (Qin *et al.*, 2007; Dardelle *et al.*, 2010; Wang *et al.*, 2010). Treatment of pollen tubes with βGlcY halts tip growth (Mollet *et al.*, 2002) and some AGP epitopes have been localized on the tube surface (Jauh and Lord, 1996; Chen *et al.*, 2007; Speranza *et al.*, 2009). The AGP epitopes recognized by the mAbs LM2 and JIM13 are deposited on the pollen tube surface of the majority of mono- and dicotyledonous species analysed to date, so are likely to be of central importance for pollen tip growth (Nguema-Ona *et al.*, 2012).

The literature indicates a possible function of AGPs in root hair development. To validate this hypothesis we analysed the role of AGPs in this process using barley rhizodermis of root hair mutants and their parent lines as a model system. We investigated the effect of βGlcY treatment on barley root hair development and the localization of 10 AGP epitopes in roots, with particular focus on the rhizodermis.

## Materials and methods

### Plant material and growing conditions

The analysis involved the following wild-type cultivars of barley (*Hordeum vulgare* L.): Dema, Diva, Karat, and Optic, along with the root hair mutants *rhl1.b*, *rhp1.a rhs1.a*, *rhs2.a*, *rhs.3a*, *and rhs4.a* (Table 2), all of which have been described by Chmielewska *et al.* (2014). Caryopses were surface sterilized by immersion in 20% household bleach and then germinated under aeroponic conditions in glass tubes sealed with Parafilm (Szarejko *et al.*, 2005) maintained under a 16h photoperiod (180 µEm^–2^ s^–1^ light) at 20°C for 5 days.

### βGlcY treatment

The Yariv reagent βGlcY [1,3,5-tris (4-β-D-glycopyranosyloxyphenylazo)-2,4,6-trihydroxy-benzene] (Biosupplies, Bundoora, Australia) stock solution (2mg ml^–1^) prepared in 0.15M NaCl was dissolved in de-mineralized water to obtain working solutions of 25 µM, 10 µM, and 1 µM. The seedlings were exposed to βGlcY in hydroponic culture for 5 days following Marzec *et al.* (2014*b*), while control sets of seedlings were grown in either de-mineralized water or in 25 µM α-D-galactosyl Yariv reagent (αGalY) (Biosupplies), prepared as described above. Three biological replicates, each comprising at least five seedlings per treatment, were included. Mean root hair lengths were based on at least 1000 root hairs measured from 15 roots, and were compared with one another using the Student’s *t-*test (*P* < 0.05).

### Immunolocalization of AGP epitopes

Root sections of length 2mm were fixed by immersion for 4h at room temperature in 50mM cacodylate buffer (pH 7.2) containing 0.5% (v/v) glutaraldehyde and 2.0% (v/v) formaldehyde. Following a 15min rinse in cacodylate buffer and two washes in distilled water, the materials were dehydrated by passage through an ethanol series (30–100%), then infiltrated with LR White resin (Sigma Aldrich, Munich, Germany), initially 33%, then 66%, and finally 100%. The samples were thereafter transferred into BEEM capsules (SPI Supplies, West Chester, USA) and polymerized at 60°C for 48h. Ultra-thin (70nm) sections and semi-thin (0.5 µm) ones were cut using an Ultracut UCT instrument (Leica, Wetzlar, Germany). The former were transferred onto copper grids for subsequent immunogold labelling while the latter were mounted on poly-L-lysine-covered slides. The anti-AGP mAbs JIM4, JIM8, JIM13-17, LM2, LM14, and MAC207 (PlantProbes, Leeds, UK) were diluted 1:20 for both the fluorescence- and immunogold-labelled detection of AGPs. The fluorescence-labelling procedure followed that of Srivastava *et al.* (2007), and was based on the use of goat anti-rat antibody conjugated with DyLight 488 fluorochrome (Thermo Scientific, Rockford, USA). Sections were analysed using a confocal laser scanning microscope (Zeiss LSM 510 META; Zeiss, Jena, Germany); cell wall autofluorescence was detected using a 364nm laser line equipped with a 385 long-pass filter, while the fluorescence of secondary antibodies was captured by an argon 488-laser equipped with a 560–615nm band pass filter. Immunogold labelling was based on the use of a goat anti-rat antibody conjugated with 10nm gold particles, as described by Teige *et al.* (1998); for ultrastructural analysis, an FEI Tecnai Sphera G^2^ (FEI, Eindhoven, The Netherlands) was used operating at 120kV.

### Whole-mount immunolabelling of AGP epitopes

The same root sections described above were used for whole-mount immunolabelling, employing the same buffers and antibody dilutions. Goat anti-rat DyLight 488 was used as a secondary antibody for fluorescence labelling. For scanning electron microscopy (SEM), the secondary antibody was goat anti-rat conjugated with 1nm gold particles. A Silver Enhancing kit (BBI Solutions, Cardiff, UK) was included, following Talbot *et al.* (2002). The signal was detected using a FESEM S 4100 device (Hitachi High-Technologies Europe GmbH, Krefeld, Germany).

## Results

### βGlcY treatment inhibited root hair development in barley

**Fig. 1.**
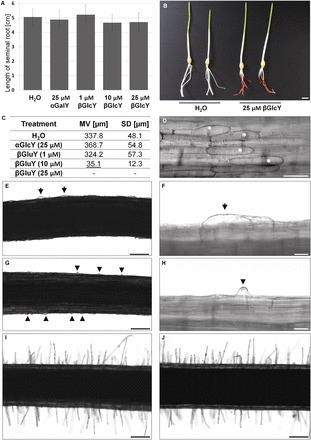
The effect of βGlcY treatment on root growth and root hair differentiation in barley cv. Karat. (A, B) Root length was not significantly influenced, while (C–J) root hair development was inhibited. (C) Root hair lengths estimated from at least 1000 root hairs sampled from 15 roots. (D) The response of root epidermal cells to 25 µM βGlcY. (E–J) Light microscopy analysis: (E,F) hairless mutant roots exposed to 25 µM βGlcY; (G–H) primordia only producing mutant roots exposed to 10 µM βGlcY; (I) treatment with 1 µM βGlcY had no effect on root hair elongation; (J) a control treatment with 25 µM αGalY. Asterisks, shorter epidermal cells; arrows, bulging cells; arrowheads, primordia; MV, mean value; SD, standard deviation. Scale bars in (B) 1cm, (D) 100 µm, (E, G, and I–J) 200 µm; (F, H) 20 µm. Underlined mean value in table indicate the statistical significance, in comparison to control conditions (Student’s t test (P < 0.05)).

There was no difference with respect to either the length or number of seminal roots formed by the parent cultivar plants in response to any of the three concentrations of βGlcY tested (Fig. 1A, B). In the presence of 25 µM βGlcY, the roots of cultivars Dema, Diva, Karat, and Optic all failed to form root hair tubes (Fig. 1C; Supplementary Table S1). Exposure to 10 µM βGlcY stopped root hair development at the primordium stage, while the 1 µM treatment had no effect on root hair length (Fig. 1C–J). In control plants treated with either demineralized water or αGalY (AGP-unreactive form of Yariv reagent), fully developed root hairs were formed, confirming the inhibitory effect of βGlcY on root hair tube elongation (Fig. 1C,J). Both light microscopy and SEM analysis showed that root hairs failed to develop on roots exposed to 25 µM βGlcY (Fig. 1D–F; Supplementay Figure S1), but the alternation of trichoblasts and atrichoblasts was maintained (Fig. 1D). A few epidermal cells bulged as a consequence of radial expansion (Fig. 1E, F).

### The presence of AGP in the barley root

**Fig. 2.**
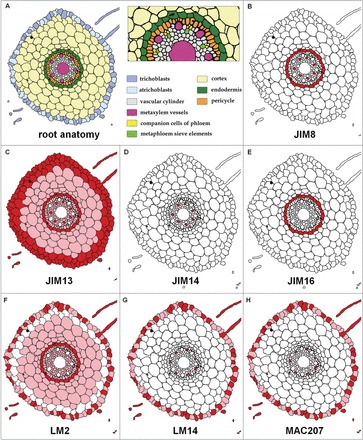
Schematic overview of AGP epitope distribution derived from transverse sections made from the mature root hair zone. (A) The organization of the root. (B–H) Abundance of the various AGP epitopes. (B) JIM8 in the endodermis and metaphloem sieve elements. (C) JIM13 was distributed throughout the root except in the metaxylem. (D) JIM14 was restricted to the metaphloem sieve elements, whereas (E) JIM16 was restricted to the endodermis. The three epitopes (F) LM2, (G) LM14, and (H) MAC207 were heterogeneously distributed in the epidermis. Signal strength indicated by colour: dark red (strong), light red (weak), and white (none).

Of the set of mAbs used to detect AGP, JIM4, JIM15, and JIM17 all failed to detect any epitopes in either transverse or longitudinal sections of the meristematic and mature root hair zone of the parent cultivars Dema, Diva, Karat, and Optic. Otherwise, epitopes were detected as follows: JIM8, endodermis and metaphloem sieve elements (Fig. 2B); JIM13, throughout the root but especially in the rhizodermis, the external layer of the cortex, the endodermis, and the metaphloem sieve elements (Fig. 2C); JIM14, only in the metaphloem sieve elements (Fig. 2D); JIM16, in the endodermis (Fig. 2E); LM2, throughout the root, except for the external cell layer in the cortex and metaxylem, and most strongly in the root hair cells and endodermis (Fig. 2F) [a similar distribution was present in the root zone in which cell differentiation was initiated (Supplementary Figure S2)]; LM14 and MAC207, in the phloem companion cells and the root epidermis, again more abundantly in the root hair cells (Fig. 1G and Table 1), especially in the differentiation zone of the root, where the difference between trichoblast and atrichoblast cell size was most apparent (Supplementary Figure S3).

**Table 1.** AGP epitopes detected in the roots of wild-type cultivars

mAb	Epidermis		Cortex	Endodermis	Pericycle	Xylem	Metaphloem sieve elements	Companion cells of phloem	Vascular cylinder
	Atrichoblasts	Trichoblasts							
JIM4	–	–	–	–	–	–	–	–	–
JIM8	–	–	–	+	–	–	+	–	–
JIM13	++	++	+	++	+	–	+	–	+
JIM14	–	–	–	–	–	–	+	–	–
JIM15	–	–	–	–	–	–	–	–	–
JIM16	–	–	–	+	–	–	–	–	–
JIM17	–	–	–	–	–	–	–	–	–
LM2	+	++	+	+	+	–	+	+	+
LM14	+	++	–	–	–	–	–	+	–
MAC207	+	++	–	–	–	–	–	+	–

### Distribution of LM2, LM14, and MAC207 epitopes in the Karat and root hairless mutant rhizodermis

**Fig. 3.**
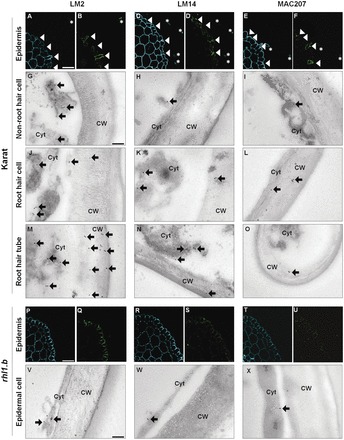
Immunolocalization of LM2, LM14, and MAC207 epitopes in the rhizodermis of barley cv. Karat and the *rhl1.b* mutant. (A, C, E, P, R, T) Autofluorescence illustrates cell patterning in the mature root hair zone. Fluorescence labelling of AGP epitopes in (B, D, F) cv. Karat and (Q, S, U) the *rhl1.b* mutant, with (B, D, F, Q, S, U, G–O, V–X) showing subcellular localization based on immunogold labelling. (A–F) Epitopes were more abundant in the trichoblasts and root hair tubes than in the atrichoblasts. (G–O) In the trichoblast cell wall, LM2, LM14, and MAC207 epitopes were only detected in the wild-type cultivars (H–X). In the root hairless mutant, the three epitopes were homogeneously distributed within the epidermis. Asterisks, root hair tubes; arrowheads, trichoblasts; arrows, gold particles; CW, cell wall; Cyt, cytoplasm. Scale bars: (A–F and P–U) 50 µm; (G–O and V–X), 100nm.

The presence of LM2, LM14, and MAC207 epitopes within the root epidermis of cv. Karat, as demonstrated by transmission electron microscopy (TEM), was consistent with the patterns obtained using confocal laser scanning microscopy (CLSM). The LM2 epitope was present throughout the epidermis, but was especially abundant in the root hairs and trichoblasts (Fig. 3A, B). In the former cell type, it was concentrated in the cell wall and cytoplasm (Fig. 3J, M). There was little accumulation in the cytoplasm of non-root hair cells, and none in the atrichoblast cell wall (Fig. 3G). Both LM14 and MAC207 epitopes were present in the cytoplasm and cell walls of root hairs (Fig. 3C–F, N, and O) and trichoblasts (Fig. 3C–F, K, and L); and in the atrichoblast cytoplasm (Fig. 3H, I). A similar analysis of the *rhl1.b* root hairless mutant (derived from cv. Karat; Table 2) showed that all three epitopes were present in the rhizodermal layer, but that there was not diverse expression of epitopes analysed (Fig. 3P–U). TEM analysis revealed that LM2, LM14, and MAC207 epitopes were evenly distributed throughout the rhizodermis, but were restricted to the cytoplasm (Fig. 3V–X); this distribution resembled that seen in the atrichoblasts of cv. Karat (Fig. 3G–I).

**Table 2.** Root hair mutants and their parent cultivar

Gene symbol	Mutant name	Parent cultivar	Phenotype
*rhl1.b*	*root hairless 1.b*	Karat	Root hairless phenotype
*rhp1.a*	*root hair primoria 1.a*	Dema	Trichoblasts produce only bulges (primordia) unable to enter tip growth
*rhs1.a*	*root hair short 1.a*	Diva	Root hairs shorter (from 40 to 90%) in comparison to those in the parent cultivar
*rhs2.a*	*root hair short 2.a*	Dema	
*rhs3.a*	*root hair short 3.a*	Karat	
*rhs4.a*	*root hair short 4.a*	Optic	

### LM2 epitope was present on the wild-type root hair tube surface

**Fig. 4.**
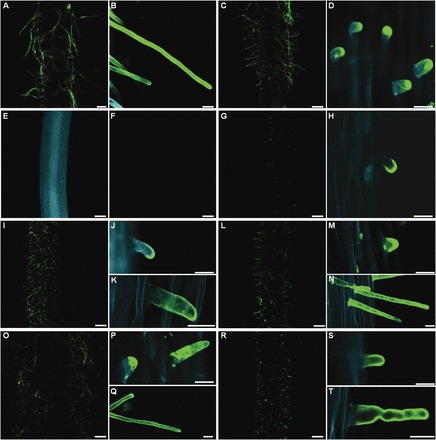
The localization of LM2 epitopes in whole-mount immunolabelled root sections of barley cultivars Karat and Dema, and the root hair mutants *rhl1.b*, *rhp1.a*, *rhs1.a*, *2.a*, *3.a*, and *4.a*. Epitope was detected (A, B) on the surface of cv. Karat root-hair tubes and (C, D) in the zone harbouring root hairs in cv. Dema. (E) Autofluorescence in *rhl1.b* and (F) the lack of any epitope on the root surface. (G, H) Clear signal in the primordia formed by *rhp1.a.* (I) Epitope in *rhs1.a,* focusing on (J) young and (K) mature root hairs. Similar comparisons are shown for (L–N) *rhs2.a*, (O–Q) *rhs3.a*, (R–T) *rhs4.a*. Scale bar in (A, C, E–G, I, L, O, R) 200 µm, and in (B, D, H, J, K, M, N, P, Q, S, T) 20 µm.

**Fig. 5.**
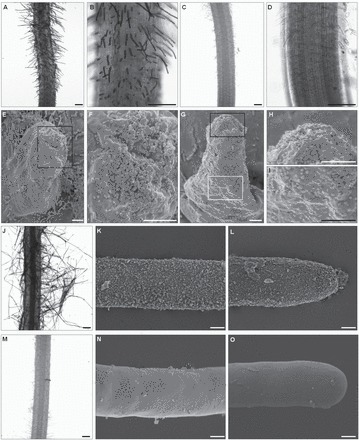
LM2 epitope distribution on the barley cv. Karat root hair surface as shown by immunogold labelling visualized by (A–D, J, M) light microscopy and (C–I, K, L, N, O) SEM. (A, B) Negative control (no primary antibody); (C, D) LM2 signal following the inclusion of gold-conjugated secondary antibody; (E) primordium; (F) detail of the primordium tip. Label strength decreased from (H) the tip to (I) the base of a growing root hair (G). (J) Root hairs displaying an even distribution of LM2 epitopes in (K) the central part and (L) the tip of fully developed root hairs. (M–O) No epitopes were detected in the negative control. Scale bar in (A–D, J, M) 200 µm, and in (E–L, N, O) 2 µm.

Of the mAbs used to investigate the distribution of AGP epitopes in the meristematic and mature root hair zone, LM2 was the only one which detected epitopes exclusively on the root surface of root hair tubes of cultivars Dema, Diva, Karat, and Optic (Fig. 4 and Supplementary Figure S4). It was even detectable on the young primordia formed during the earliest stages of root hair development (Supplementary Figure S4). At the primordium stage, the epitopes were restricted to the tip of the outgrowth (Fig. 4D), but later they became homogeneously distributed along root hair tubes (Fig. 4B; Supplementary Figure S4). SEM analysis of preparations labelled with a gold-conjugated secondary antibody confirmed the deposition pattern of LM2 epitope AGPs. Even under light microscopy, the presence of LM2 epitope was observed in cv. Karat on the surface of primordia and root hair tubes (Fig. 5A, B, J), whereas in the negative control no signal was detected (Fig. 5C,D,M). SEM observations confirmed deposition of LM2 epitope on the surface of primordia (Fig. 5E, F) and young root hairs (Fig. 5G–I); moreover, LM2 was still present on the tip of the mature root hair (Fig. 5K, L), whereas in the negative control no LM2 was observed on the root surface (Fig. 5N, O).

### Localization of LM2 epitope on the root surface in root hair mutants

Neither SEM or CLSM was able to detect the presence of LM2 epitope on the roots of the *rhl1.b* root hairless mutant (Fig. 4F), while in the *rhp1.a* mutant (which produces only primordia; Table 2), epitopes were restricted to the tip of outgrowths (Fig. 4G, H). In the four non-allelic short root hair mutants *rhs1.a*, *2.a*, *3.a*, and *4.a* (Table 2) the localization of LM2 epitopes was comparable to that observed in the wild-type root; they were distributed over the whole surface of the root hair tubes formed by fully developed root hairs (Fig. 4I–T).

## Discussion

### βGlcY inhibits root hair development

This research has shown that root hair development is quantitatively inhibited by the concentration of βGlcY present in the growing medium. A level of 25 µM suppressed primordium formation and therefore blocked root hair formation at an early stage, while a level of 10 µM was insufficient to prevent primordium formation, but sufficient to halt the elongation of the root hair tubes. In *Arabidopsis* roots, βGlcY treatment results in a significant degree of radial cell expansion in the rhizodermis, causing the tissue to bulge outwards (Willats and Knox, 1996; Ding and Zhu, 1997). At the subcellular level, this phenomenon reflects a disorganization of the cortical microtubules in the epidermis (Nguema-Ona *et al.*, 2007). The inhibitory effect of βGlcY on pollen tube tip growth is well established (Mollet *et al.*, 2002). In both lily (*Lilium longiflorum*) and *Annona cherimola*, pollen tube elongation is compromised (Jauh and Lord, 1996; Mollet *et al.*, 2002); however, in *Aquilegia eximia*, *Lycopersicon pimpinellifolium*, and tobacco (*Nicotiana tabacum*), this is not the case (Mollet *et al.*, 2002), and *Arabidopsis* pollen is completely prevented from germinating (Lennon and Lord, 2000). βGlcY treatment enhances the level of cytosolic Ca^2+^ in both lily pollen tubes (Roy *et al.*, 1999) and cultured tobacco cells (Pickard and Fujiki, 2005). Both changes in cytosolic Ca^2+^ level and cytoskeleton organization are important parameters during the formation of primordia and the elongation of root hair tubes (Schiefelbein *et al.*, 1992; Park and Nebenführ, 2011). With respect to the inhibitory effect of βGlcY on barley root hair development, therefore, it seems probable that AGPs regulate the organization of the cytoskeleton and the cytosolic Ca^2+^ level.

No βGlcY effect was observable during the early stage of rhizodermal cell differentiation, specifically when the alternation of trichoblasts and atrichoblasts is first apparent (Marzec *et al.*, 2013). Since the highest concentration of βGlcY applied (sufficient to convert a wild-type into a hairless phenotype) did not modify this alternation, the implication was that the localization of AGPs on the root surface had no influence over rhizodermis patterning; rather, this must depend on asymmetric daughter cell elongation following symmetrical cell division (Marzec *et al*., 2013 2014a). Given that the inhibitory effect of βGlcY on the elongation of the *Arabidopsis* root (Willats and Knox, 1996) was not reproduced in barley, it would appear that species-distinct mechanisms must underlie the elongation of rhizodermal cells. However, in the presence of 25 µM βGlcY, some of the barley root cells did bulge outwards, implying some degree of cytoskeletal disorganization similar to that which occurs in the *Arabidopsis* rhizodermis (Nguema-Ona *et al.*, 2007).

### The distribution of AGP in the barley root

Variation in patterns of deposition of AGP epitopes between species has hindered the allocation of function to the individual classes of these proteins. Epitopes recognized by neither JIM4, JIM15, nor JIM17 were detectable in barley; similarly, in onion and pea, JIM4 epitopes are not present (Casero *et al.*, 1998), while JIM15 and JIM17 epitopes are lacking in *Benincasa hispida* (wax gourd; Xie *et al.*, 2011) (Table 3). In contast, JIM15 epitopes are ubiquitous in the carrot (*Daucus carota*) root (Knox *et al.*, 1991), while those recognized by JIM4 are present in the protoxylem/pericycle of carrot and radish (Casero *et al.*, 1998). The tissue distribution of JIM13 epitopes is particularly variable: they are seen throughout the barley root but are only detectable in the developing xylem in carrot (Dolan and Roberts, 1995) and in the pericycle and protophloem sieve elements in maize (*Zea mays*; Samaj *et al.*, 1998); they are completely missing in *B. hispida* (Xie *et al.*, 2011). Although JIM13 epitope was abundant in the rhizodermis, endodermis, and metaphloem sieve elements, no specific developmental process could be assigned to the class of AGP harbouring β-D-GlcA-(1,3)-α-D-GalA-(1,2)-α-L-Rha (Yates and Knox, 1994; Yates *et al.*, 1996).

**Table 3.** AGP epitopes detected in the roots of various plant species

mAbs	Recognized epitope	Epitope expression in roots
JIM4	β-D-GlcA-(1,3)-α-D-GalA-(1,2)-α-L-Rha (Yates *et al.*, 1996)	Carrot: protoxylem, pericycle (Knox *et al.*, 1989 1991; Casero *et al.*, 1998) Radish: individual pericycle cells (Casero *et al.*, 1998) Onion, pea: lack of expression in all root tissues (Casero *et al.*, 1998) Barley: lack of expression in all root tissues (this work)
JIM8	Unknown	Maize: protophloem sieve elements (Samaj *et al.*, 1998) Wax gourd: protoxylem (Xie *et al.*, 2011) Barley: endodermis and metaphloem sieve elements (this work)
JIM13	β-D-GlcA-(1,3)-α-D-GalA-(1,2)-α-L-Rha (Yates and Knox, 1994; Yates *et al.*, 1996)	*Arabidopsis*: xylem (Dolan *et al.*, 1995); root cap and all root apex cells (Vicre *et al.*, 2005) Carrot: early stage of xylem development (Knox *et al.*, 1991) Maize: pericycle, protophloem sieve elements, companion cells, root cap (Samaj *et al.*, 1998) Wax gourd: lack of expression in all root tissues (Xie *et al.*, 2011) Barley: all root cells, stronger in epidermis, endodermis, and metaphloem sieve elements (this work)
JIM14	Unknown	*Arabidopsis*: all root cells, stronger in metaphloem sieve elements (Dolan and Roberts, 1995) Carrot: all root cells (Knox *et al.*, 1991) Wax gourd: lack of expression in all root tissues (Xie *et al.*, 2011) Barley: metaphloem sieve elements (this work)
JIM15	Unknown	Carrot: all root cells, except epidermis (Knox *et al.*, 1991) Wax gourd: lack of expression in all root tissues (Xie *et al.*, 2011) Barley: lack of expression in all root tissues (this work)
JIM16	Unknown	Carrot: all cells in root meristem (Knox *et al.*, 1989) Wax gourd: all root cells, weaker in cortex and parenchyma (Xie *et al.*, 2011) Barley: endodermis (this work)
JIM17	Unknown	Wax gourd: lack of expression in all root tissues (Xie *et al.*, 2011) Barley: lack of expression in all root tissues (this work)
LM2	β-Linked GlcA (Smallwood *et al.*, 1994)	*Arabidopsis*: epidermis, weaker in trichoblasts (Andeme-Onzighi *et al.*, 2002) Maize: surface of root hair tubes (Samaj *et al.*, 1999) Wax gourd: all root cells, except root epidermis (Xie *et al.*, 2011) Barley: all cells except xylem, diverse expression in epidermal and parenchyma cells (this work)
LM14	Arabinose- and galactose-enriched carbohydrate chains (Moller *et al.*, 2008)	Wax gourd: all root cells, stronger in epidermis (Xie *et al.*, 2011) Barley: companion cells of phloem and diverse expression in epidermis (this work)
MAC207	β-GlcA-(1,3)-α-GalA-(1,2)-Rha (Bradley *et al.*, 1988; van den Bosch *et al.*,1989)	*Arabidopsis*: root cap and all root apex cells (Vicre *et al.*, 2005) Carrot: all cells in root meristem (Knox *et al.*, 1989) Wax gourd: lack of expression in all root tissues (Xie *et al.*, 2011) Barley: companion cells of phloem and diverse expression in epidermis (this work)

JIM8, JIM14, and JIM16 mAbs can each serve as a marker for particular root tissues in barley: the former two for metaphloem sieve elements, and the latter one for the endodermis. JIM8 epitopes are also detected in maize root immature and mature sieve elements (Samaj *et al.*, 1998) and in the *B. hispida* protoxylem (Xie *et al.*, 2011) (Table 3). Thus JIM8 epitope AGPs may be involved in the differentiation of two types of vascular tissue in both mono- and dicotyledonous species. JIM14 epitope localization is interspecifically rather variable (Knox *et al.*, 1991; Dolan and Roberts, 1995; Xie *et al.*, 2011). In barley, they were restricted to the metaphloem sieve elements, while in *Arabidopsis* they are abundant throughout the root (Dolan *et al.*, 1995) (Table 3). JIM16 epitopes were observed solely in the root endodermis of barley, while in both carrot (Knox *et al.*, 1989) and *B. hispida* (Xie *et al.*, 2011) they are distributed throughout the root; the inference is that these epitopes are unlikely to be generally involved in cell differentiation.

### The localization of LM2, LM14, and MAC207 epitopes in the barley rhizodermis

LM2 targets the β-linked GlcA molecule present in the AGP polysaccharide moiety (Smallwood *et al.*, 1994), and was particularly abundant in trichoblasts/root hair tubes. In *Arabidopsis*, LM2 epitopes are present in the rhizodermis (especially in the trichoblasts), while in the *reb1* mutant they are confined to atrichoblasts (Andeme-Onzighi *et al.*, 2002). The implication is that AGPs containing GlcA are required for root hair differentiation in a wide range of species. In both maize and sundew (*Drosera capensis*), LM2 epitopes are deposited in the root epidermis, parenchyma, and cortex (Samaj *et al.*, 2000). TEM analysis has revealed a preferential localization within the cytoplasm endomembrane system, in association with the endoplasmic reticulum, Golgi apparatus, and tonoplast in both species (Samaj *et al.*, 2000). The present experiments have demonstrated the presence of LM2 epitopes in various tissues of the barley root, and of particular interest is their distribution between trichoblasts and atrichoblasts and among the various parenchyma layers. While they were largely restricted to the cell wall of root hairs and tubes, some were also present in the atrichoblast cytoplasm. In root hair tubes, they were associated with vesicles, just as they are in both maize and sundew (Samaj *et al.*, 2000). At an early stage of root hair formation, when the cell alternation pattern is first visible (Marzec *et al.*, 2013), more abundant LM2 epitope was present in the cytoplasm of nascent trichoblasts. The lack of LM2 epitope in the external layer of the cortex, in comparison to its low level presence in the cytoplasm of other layers, can be explained by a combination of differential expression of the gene encoding the protein recognized by LM2, variable post-transcriptional modification of proteins (Seifert *et al.*, 2014), and the influence of symplasmic communication on the transport and location of AGPs among cells of the same tissue (Marzec and Kurczynska, 2014). AGPs are known to contribute to signalling and cell-to-cell communication, and the presence of the LM2 epitope in the layer of cells located below the root epidermis could probably interfere with communication between adjacent rhizodermal cells. As the greater abundance of the LM2 epitope coincided with an early stage of barley root hair development, it is possible that the relevant AGPs are transported to the cell wall only in root hair cells. In maize, the finding that LM2 epitope is deposited on the surface of root hair tubes has been suggested to imply that these AGPs have a function in root hair elongation (Samaj *et al.*, 1999). The same epitopes were present on the barley root surface from the earliest stage of root hair development, although unlike in maize, they remained at the tip of the mature root hairs. In both the *rhp1.a* mutant and the four non-allelic *rhs* mutants, the epitopes remained detectable on the surface of primordia/root hair tubes. A consistent hypothesis is therefore that the presence of certain AGP epitopes on the root hair surface is required for the development of the root hair. Because there was no discernible effect of fixation or dehydration on the distribution of the LM2 epitope, the likelihood is that the relevant AGP remained anchored to GPI (and so to the plasma membrane), rather than being secreted into the extracellular matrix.

A similar distribution of epitopes applied to the AGPs recognized by LM14, a mAb which targets arabinose- and galactose-enriched carbohydrate chains (Moller *et al.*, 2008), and MAC207, which targets β-GlcA-(1,3)-α-GalA-(1,2)-Rha (Bradley *et al.*, 1988; van den Bosch *et al.*, 1989). Although the abundance of these epitopes was lower than for those recognizing LM2, there remained a clear difference between their abundance on trichoblasts/root hair tubes and on atrichoblasts. LM14 epitopes are present throughout the *B. hispida* root and particularly in the rhizodermis, but no difference in abundance appears to exist between root hair and non-root hair cells (Xie *et al.*, 2011) (Table 3). In barley, the presence of LM14 epitopes was restricted to the root epidermis and phloem sieve elements, a finding which allows this mAb to be informative as a marker for these tissues. More specifically, the epitope was detected in the trichoblast cell wall, all the way from the earliest stage of root hair formation to the final, mature stage. *B. hispida* roots lack any MAC207 epitopes (Xie *et al.*, 2011), whereas the antigen is present in both *Arabidopsis* and carrot root cells (Knox *et al.*, 1989; Vicre *et al.*, 2005). In barley, the distribution of MAC207 epitope overlapped that of LM14 (Table 3). The pattern of LM2, LM14 and MAC207 epitope deposition in barley suggests a coincidence of epitope transport/localization and rhizodermal cell differentiation.

In contrast to the wild-type cultivars, in which the presence of all three epitopes was marginal in atrichoblasts but substantial in trichoblasts, in the root hairless mutant, LM2, LM14, and MAC207 epitopes were dispersed at a low level of abundance throughout the rhizodermis. This observation is fully consistent with the downregulation of a gene encoding AGP in the mutant root, whereas no such differential transcription could be observed between mutants producing primordia and the parent cultivar (Kwasniewski *et al.*, 2010). The present immunolocalization experiments in mutants generating a distinct root hair phenotype have led to a suggested role for each of the three classes of AGP during the early stage of root hair development and have established correlations between their cellular localization and rhizodermal cell specialization.

## Supplementary material

Supplementary data can be found at *JXB* online.

Supplementary Table S1. The effect of βGlcY treatment on root hair tube elongation in barle**y** cultivars Dema, Diva, and Optic.

Supplementary Figure S1. SEM analysis of βGlcY-induced inhibition of root hair elongation in barle**y** cv. Dema.

Supplementary Figure S2. CLSM analysis of LM2 epitope deposition in barley cv. Karat root.

Supplementary Figure S3. Localization of MAC207 epitopes in barley cv. Karat roots as visualized by CLSM.

Supplementary Figure S4. LM2 epitopes on the barley cv. Dema root surface as visualized by CLSM.

## Funding

The research was carried out in the framework of the two Polish National Science Centre grants 2011/01/M/NZ2/02979 and 2013/08/T/NZ3/00811. M. Marzec was supported by a Foundation for Polish Science scholarship (START 071/2014).

## Supplementary Material

Supplementary Data
