# Supplementary material for: Arabinogalactan proteins are involved in root hair development in barley
Source: J Exp Bot. 2014 Dec 1;66(5):1245–57. doi: 10.1093/jxb/eru475 (PMC4339589; doi:10.1093/jxb/eru475)
Supplement: Supplementary Data [file supp_66_5_1245__index.html]

Arabinogalactan proteins are involved in root hair development in barley — Arabinogalactan proteins are involved in root hair development in barley — Supplementary Data 

# Arabinogalactan proteins are involved in root hair development in barley

## Supplementary Data

Data files

**Files in this Data Supplement:**

- Supplementary Data - Supplementary Data
