# Supplementary material for: Arabinogalactan proteins are involved in root hair development in barley
Source: J Exp Bot. 2014 Dec 1;66(5):1245–57. doi: 10.1093/jxb/eru475 (PMC4339589; doi:10.1093/jxb/eru475)
Supplement: Supplementary Data [file supp_eru475_jexbot137489_file001.pdf]

Arabinogalactan proteins are involved in root hair development in barley

Marek Marzec, Iwona Szarejko, and Michael Melzer

SUPPLEMENTARY DATA

Supplementary Table S1. The effect of βGlcY treatment on root hair tube elongation in barley cvs ‘Dema’, ‘Diva’ and ‘Optic.

	**‘Dema’**	**‘Diva’**	**‘Optic’**
**H_2O**	409.1 µm (±41.6)	386.1 µm (±39.4)	356.7 µm (±38.2)
**αGlcY (25 µM)**	389.8 µm (±48.1)	396.4 µm (±45.1)	379.3 µm (±45.7)
**βGluY (1 µM)**	378.6 µm (±50.5)	376.5 µm (±40.9)	374.1 µm (±48.6)
**βGluY (10 µM)**	29.1 µm (±12.8)	42.8 µm (±16.6)	27.1 µm (±13.9)
**βGluY (25 µM)**	-	-	-

Mean root hair lengths were based on at least 1,000 root hairs measured from 15 roots in 3 biological repeats. Underlined mean values in table indicate the statistical significance, in comparison to control conditions (Student's *t* test ($P < 0.05$)).

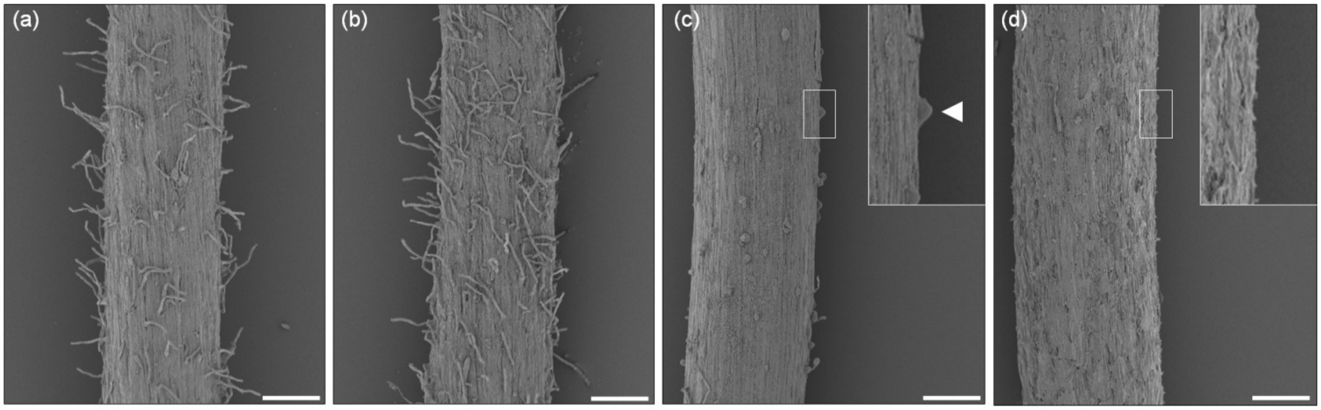

Supplementary Figure S1. SEM analysis of BGlcY-induced inhibition of root hair elongation in cv. 'Dema' plant exposed to (a) 25 μM αGalY, (b) 1 μM βGlcY, (c) 10 μM βGlcY, and (d) 25 μM βGlcY. Arrowhead indicates primordium. Scale bar 200 μm.

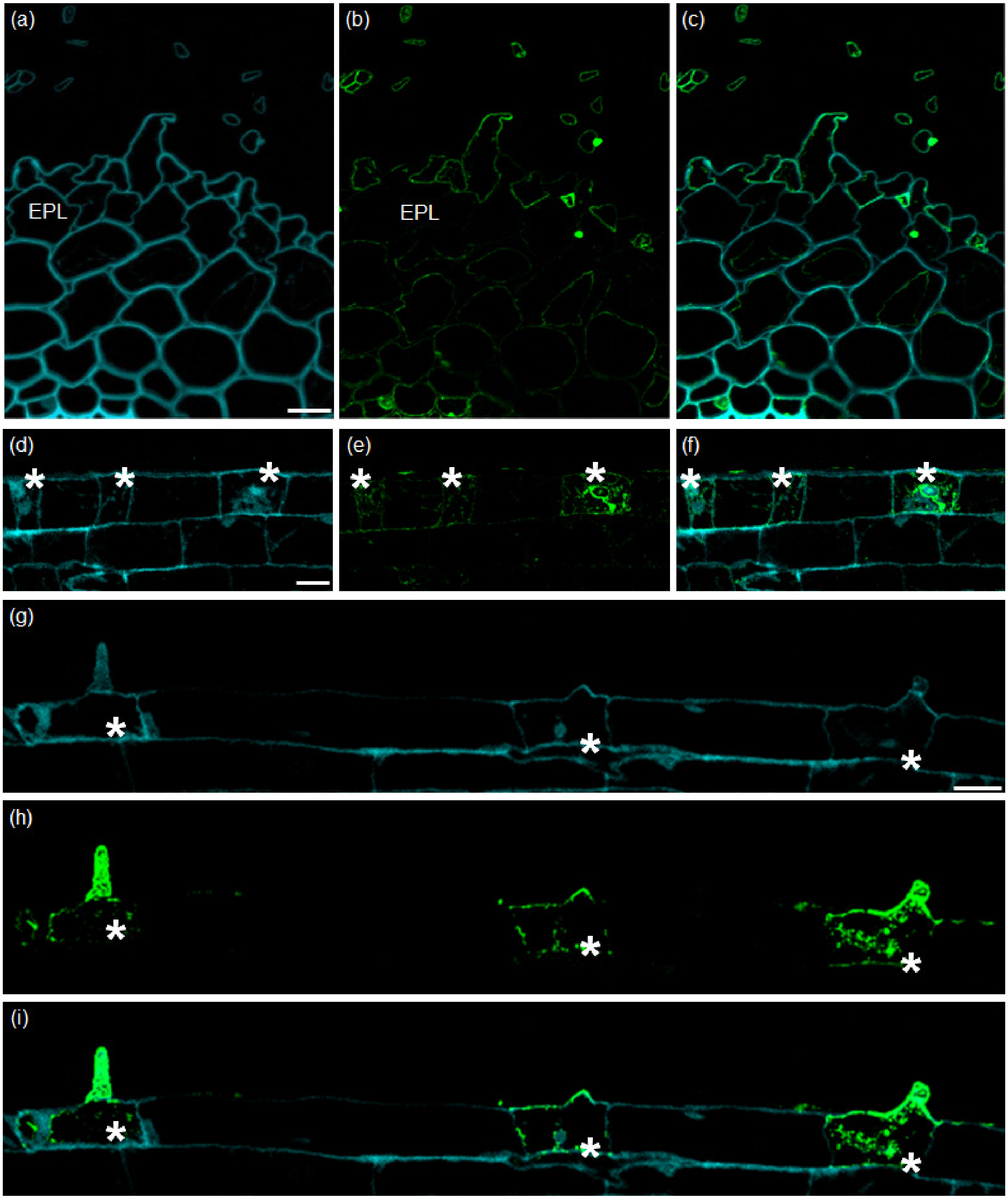

Supplementary Figure S2. CLSM analysis of LM2 epitope deposition (marked in green) in the cv. ‘Karat’ root. (a-c) Transverse section of the mature root hair zone, (d-f) longitudinal section of the differentiation zone, and (h,i) nascent root hairs. Asterisks indicate trichoblasts. EPL: external parenchyma layer. Scale bar 20 µm

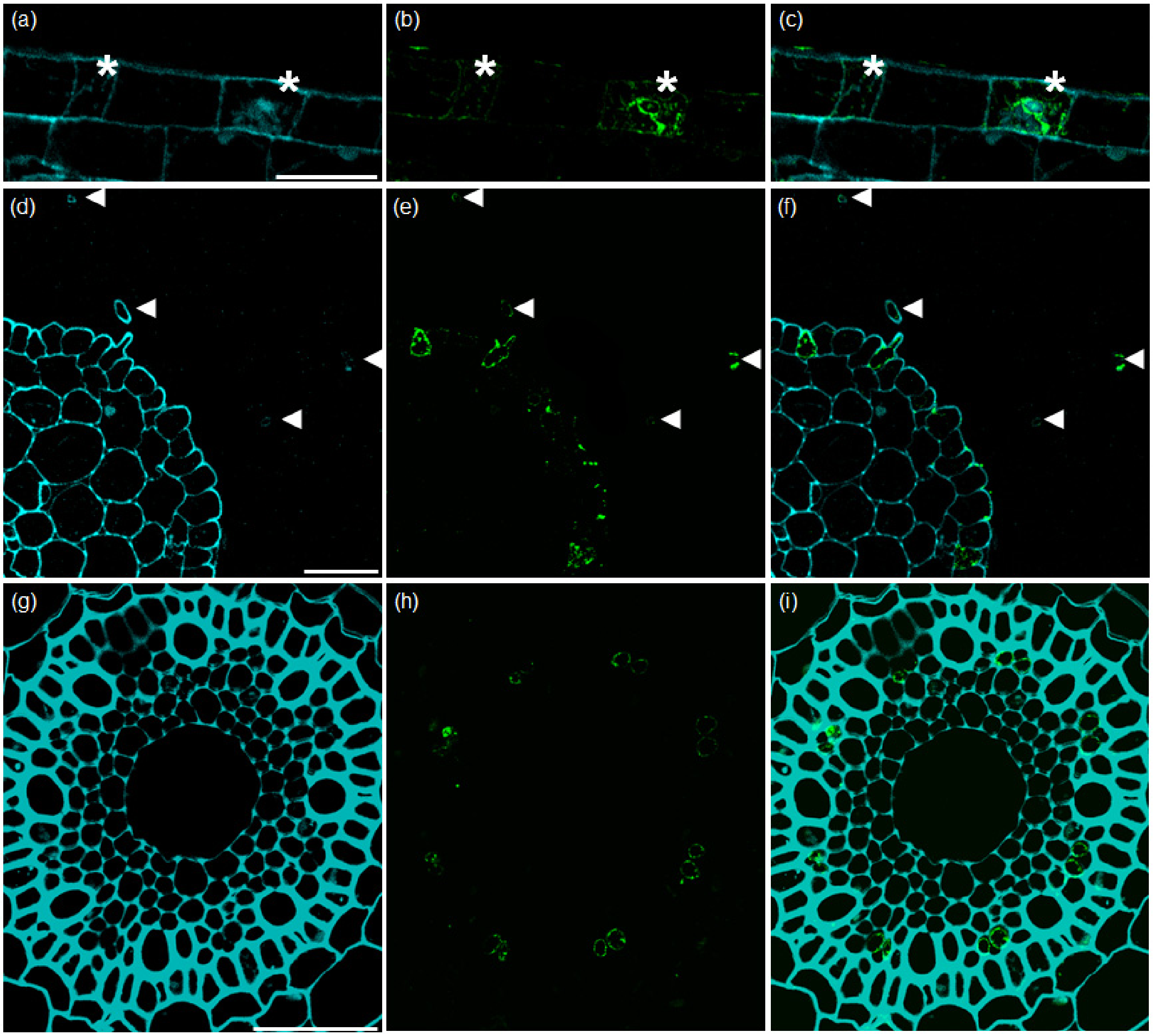

Supplementary Figure S3. Localization of MAC207 epitopes in cv. 'Karat' roots, as visualized by CLSM. (a-c) A longitudinal section of the differentiation zone revealed a concentration of epitopes in the trichoblasts, while (d-f) a traverse section showed their presence in root hair cells and tubes, and (g-i) in phloem sieve elements. Asterisks indicate trichoblasts, and arrowheads root hair tubes. Scale bar 50µm.

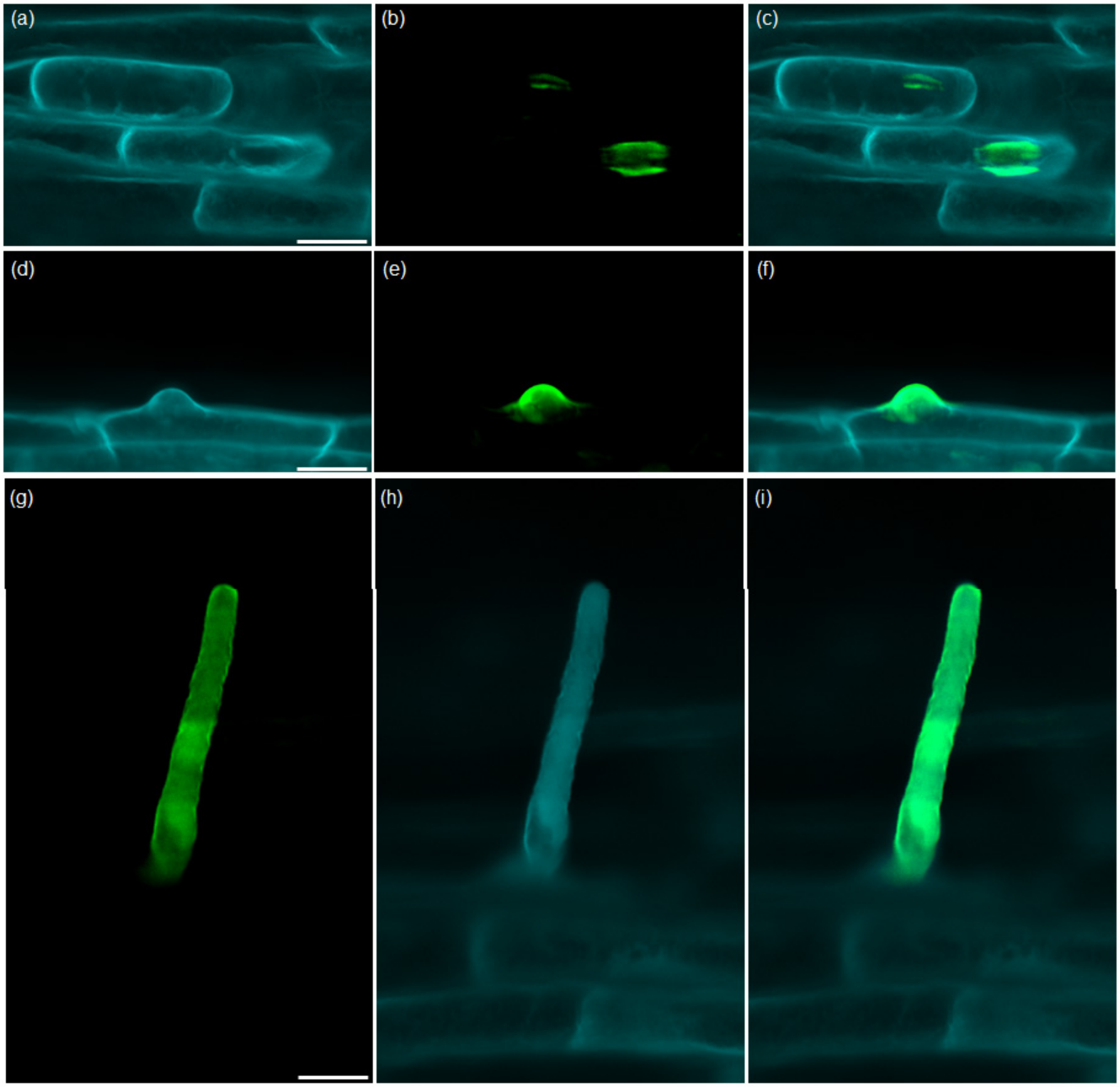

Supplementary Figure S4. LM2 epitopes on the cv. ‘Dema’ root surface as visualized by CLSM. Epitopes present (a-c) in the root hair extracellular matrix at an early stage of root hair differentiation, (d-f) in the developed primordium, and (g-i) in the root hair tubes. Scale bar 20 µm.
